# Mass spectrometry analysis of gut tissue in acute SIV-infection in rhesus macaques identifies early proteome alterations preceding the interferon inflammatory response

**DOI:** 10.1038/s41598-022-27112-y

**Published:** 2023-01-13

**Authors:** A. R. Berard, T. Hensley-McBain, L. Noël-Romas, K. Birse, M. Abou, G. Westmacott, S. McCorrister, J. Smedley, Nichole R. Klatt, Adam D. Burgener

**Affiliations:** 1 Department of Obstetrics and Gynecology, University of Manitoba, Winnipeg, Canada; 2 Center for Global Health and Diseases, Department of Pathology, Case Western Reserve University, 10900 Euclid Ave., Cleveland, OH 44106 USA; 3 McLaughlin Research Institute for Biomedical Sciences, Great Falls, WA USA; 4 Department of Pharmaceutics, University of Washington, Seattle, WA USA; 5 Public Health Agency of Canada, Winnipeg, Canada; 6 Division of Pathobiology and Immunology, Oregon National Primate Research Center, Oregon Health and Science University, Beaverton, Oregon USA; 7 Division of Surgical Outcomes and Precision Medicine Research, Department of Surgery, University of Minnesota, 420 Delaware St. SE, MMC 195, Minneapolis, MN 55455 USA; 8 Department of Medicine Solna, Karolinska Institutet, Stockholm, Sweden

**Keywords:** HIV infections, Immunology, Systems biology

## Abstract

HIV infection damages the gut mucosa leading to chronic immune activation, increased morbidities and mortality, and antiretroviral therapies, do not completely ameliorate mucosal dysfunction. Understanding early molecular changes in acute infection may identify new biomarkers underlying gut dysfunction. Here we utilized a proteomics approach, coupled with flow cytometry, to characterize early molecular and immunological alterations during acute SIV infection in gut tissue of rhesus macaques. Gut tissue biopsies were obtained at 2 times pre-infection and 4 times post-infection from 6 macaques. The tissue proteome was analyzed by mass spectrometry, and immune cell populations in tissue and blood by flow cytometry. Significant proteome changes (*p* < 0.05) occurred at 3 days post-infection (dpi) (13.0%), 14 dpi (13.7%), 28 dpi (16.9%) and 63 dpi (14.8%). At 3 dpi, proteome changes included cellular structural activity, barrier integrity, and activation of epithelial to mesenchymal transition (EMT) (FDR < 0.0001) prior to the antiviral response at 14 dpi (IFNa/g pathways, *p* < 0.001). Novel EMT proteomic biomarkers (keratins 2, 6A and 20, collagen 12A1, desmoplakin) and inflammatory biomarkers (PSMB9, FGL2) were associated with early infection and barrier dysfunction. These findings identify new biomarkers preceding inflammation in SIV infection involved with EMT activation. This warrants further investigation of the role of these biomarkers in chronic infection, mucosal inflammation, and disease pathogenesis of HIV.

## Introduction

Antiretroviral therapy (ART) can keep viral replication at bay for people diagnosed with HIV, leading to an increase in life expectancy. However, even with early treatment, infected individuals do not live as long as their HIV-uninfected counterparts and their quality of life is drastically decreased^1^. HIV-infected individuals show premature signs of aging, such as cardiovascular diseases, neurocognitive disorders, metabolic or bone abnormalities, and cancers, all of which are normally associated with much older individuals. These diseases are thought in part to be caused by a chronic inflammatory state due to infection, which has been termed ‘inflammaging’ or in the case of HIV infected individuals ‘inflammAIDS’^2^. ‘InflammAIDS’ linking to pathogenesis, morbidities, and mortalities, has been well documented irrespective of whether the individual is on ART.

The mechanisms of inflammation during infection are most likely multifaceted, including direct response to the virus, CMV reactivation, and microbial or microbial product translocation^3^, and are not limited to HIV infections. In intestinal diseases (Crohn’s, celiac, or inflammatory bowel disease), inflammation is linked to a decreased mucosal layer, increased epithelial permeability and bacterial product translocation^4^. Barrier integrity damage is a key factor in the development and progression of intestinal inflammation of these diseases^5^ as well as during infection with HIV^3^. The kinetics and drivers of how inflammation and barrier damage is established in the gut is currently a topic of investigation, and we have recently shown that the epithelial barrier is disturbed prior to viral peak and associated inflammation^6^.

The intestinal mucosal barrier has several interconnected components that are utilized to prevent infection and maintain homeostasis within the gut, including the physical anatomy of the barrier, the immune system, soluble factors, and interactions between the epithelial barrier with the local microenvironment and microbiome. There are numerous markers of barrier integrity and function that can be used to assess gut disruption, including intestinal fatty acid binding proteins (IFABP), glutathione s-transferases, tight junction proteins (such as zonulin), and translocation of large molecular probes through the paracellular intestinal pathway which are associated with inflammation^5^. Even after HIV treatment and viral suppression, gut barrier integrity is not restored and thought to be the major contributor to chronic inflammation with the continued exposure to microbial products^7^. However, the processes of mucosal dysfunction are not completely understood.

Recent studies on natural SIV-hosts, African green monkeys, which are able to avoid pathogenesis despite SIV infection, showed that activation of gut tissue wound healing pathways are associated with preventing epithelial barrier damage, indicating intestinal dysfunction is an underlying cause of pathogenesis^8,9^. Therefore, defining molecular mechanisms in the gut during virus infection that contribute to host pathogenesis could potentially lead to biological targets to restore gut dysfunction and decrease chronic inflammation.

Here, using the SIV infection model of rhesus macaques, we performed mass spectrometry-based analysis to investigate the proteome changes in colon and rectal tissue samples during early infection to better understand these mechanisms and to identify early biomarkers of gut dysfunction.

## Results

**Figure 1:**
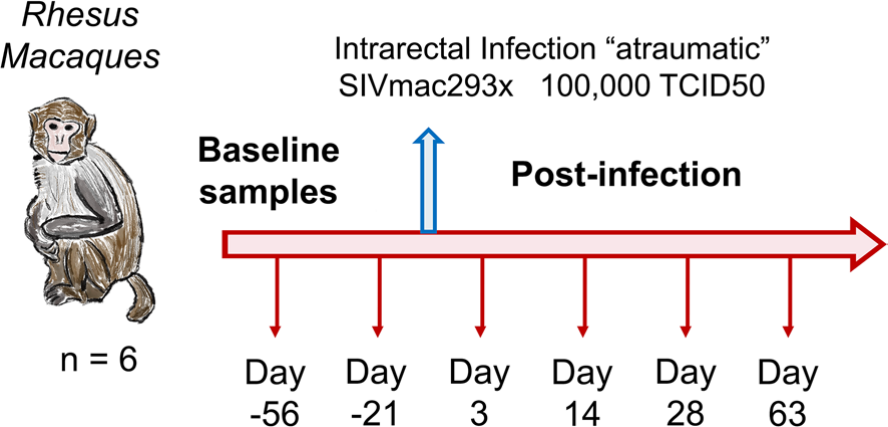
Experimental study design. Six rhesus macaques were infected with SIVmac293x by intra-rectal challenge. Colon, rectal and lymph node biopsies, as well as blood samples were collected at each time point indicated. Proteomics was performed for colon and rectal biopsies, and all 4 sample types were assessed by flow cytometry for immune cell marker expression.

Rhesus macaques (n = 6) were prospectively studied pre- and post-infection with SIV (Fig. 1). Colon biopsies, rectal biopsies, cytobrushes, and blood samples were harvested two times pre-infection (Days-56 and -21), and four times during acute infection [3, 14, 28 and 63 days post-infection (dpi)].

**Figure 2:**
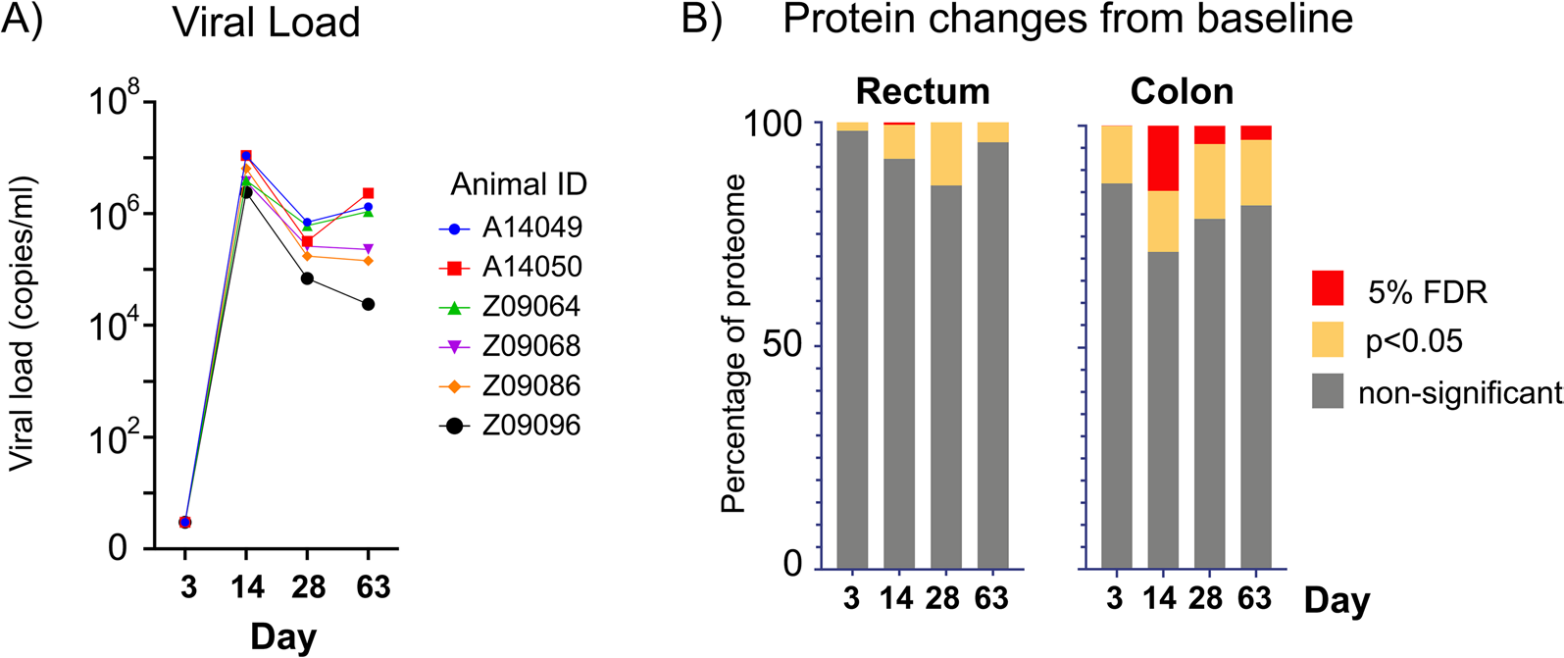
Viral load kinetics and gut proteome changes during acute SIV_MAC239x_ infection in rhesus macaques. (**a**) Viral load peaked at day 14 post-SIV infection (average 6.4 × 10^7^ copies/ml), which corresponded with an increase in (**b**) host proteome changes in both colon and rectal tissues. Total number of proteins significantly changed (5% FDR) from baseline (day-56, -21) during peak viremia at day 14 were 243/1650 (14.7%) and 13/2278 (0.5%) for colon and rectum, respectively. Increased proteome alterations were observed in the colon than the rectum post-infection.

In the colon, a total of 1650 proteins were identified at all time points, for all macaques, that passed coefficient of variation (CoV < 25%). Of these, 1 (0.6%), 243 (14.7%), 68 (4.1%) and 53 (3.2%) proteins were significantly altered (FDR 5%) at 3, 14, 28 and 63 dpi, respectively, in comparison to the average baseline values (day-56 and -21) (Supplementary Fig. 1a, Supplementary Table 1). The most robust proteomic response (14 dpi) correlated with peak viremia (Fig. 2). A total of 2278 proteins were identified in the rectal biopsies that passed coefficient of variation (CV < 25%) (Supplementary Fig. 1b, Supplementary Table 2). Of these only 13 proteins (0.6%) were significantly altered (FDR 5%) at 14 dpi in rectal biopsies. Therefore, proteome alterations were more pronounced in the colon in comparison to the rectum.

### Proteomic analysis of acute SIV infection

**Figure 3:**
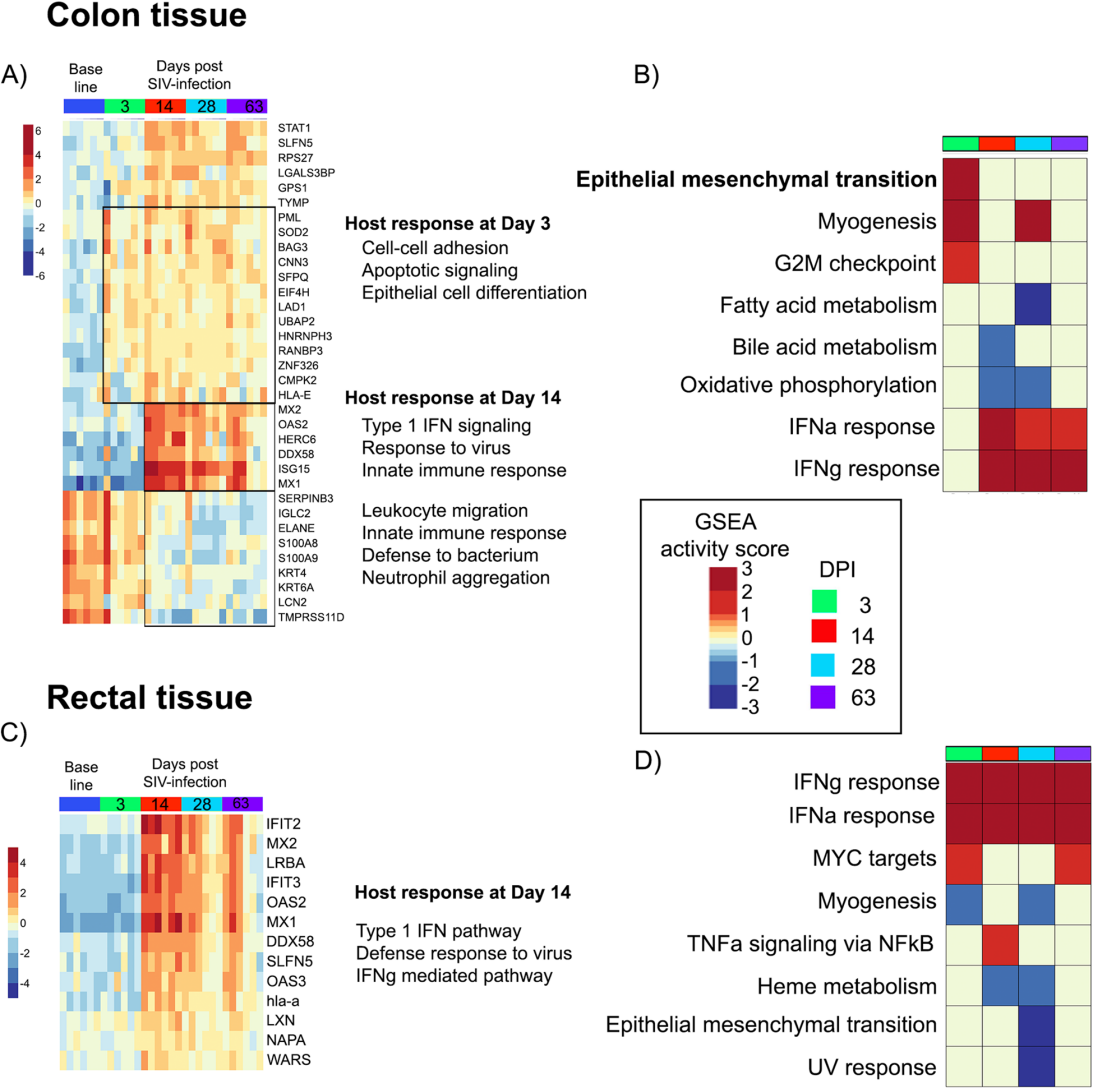
Proteome alterations in gut tissue of rhesus macaques after SIV infection. (**a**) Heatmap of significant (5% FDR) proteome changes in the colon over time during SIV infection. Changes at day 3 included proteins that function in cell–cell adhesion, indicating structural remodeling. By Day 14, biological functions related to virus defense were activated, including type 1 IFN pathway, innate immune response, and defense response to virus. (**b**) Gene Set Enrichment Analysis (GSEA) using the Hallmark Gene Database shows kinetics of proteome pathways altered during acute SIV infection. Pathways shown are those < FDR of 5%. At Day 3, epithelial to mesenchymal transition and myogenesis were activated, while IFN responses were activated at Day 14, coupled with a reduction in fatty acid metabolism and oxidative phosphorylation. (**c**) Rectal tissue proteome changes were pronounced at Day 14 post-SIV infection, showing activation of the type 1 IFN pathway and defense response to virus (**d**). GSEA of the rectal proteins showed activation of coagulation at day 3, with activation of IFN responses starting at Day 14.

We performed cluster analysis of proteins significantly altered post-infection in colon and rectal tissue, showing distinct temporal changes in both compartments (Fig. 3). In the colon, pathway analysis indicated that proteins related to cell-to-cell adhesion were significantly altered (adj.*p* = 0.0005) as early as 3 dpi (Fig. 3a). By 14 dpi, during peak viremia, activation of IFN (adj.*p* = 2.63E−6) and response to virus (adj.*p* = 1.35E−4) pathways were observed. A similar activation of the IFN signaling pathway was also observed in rectal tissue by 14 dpi (Fig. 3c, *p* = 9.43E−11). Gene set enrichment analysis (GSEA) performed on data from both compartments showed an increase in IFN responses by 14 dpi (FDR < 0.0005), which continued up to 63 dpi (Fig. 3b,d). The colon showed a stronger proteome response to infection on 3 dpi in comparison to the rectum (Fig. 3b). Mitochondrial functions (oxidative phosphorylation, fatty acid metabolism) were decreased (adj.*p* < 0.05) early in the colon and later in the rectum. However, only 26–29% of the proteins involved in these pathways overlapped between intestinal compartments. Interestingly, myogenesis (adj.*p* = 0.0008) and epithelial to mesenchymal transition (EMT) (adj.*p* < 0.0001) pathways were increased at 3 dpi in the colon.

**Figure 4:**
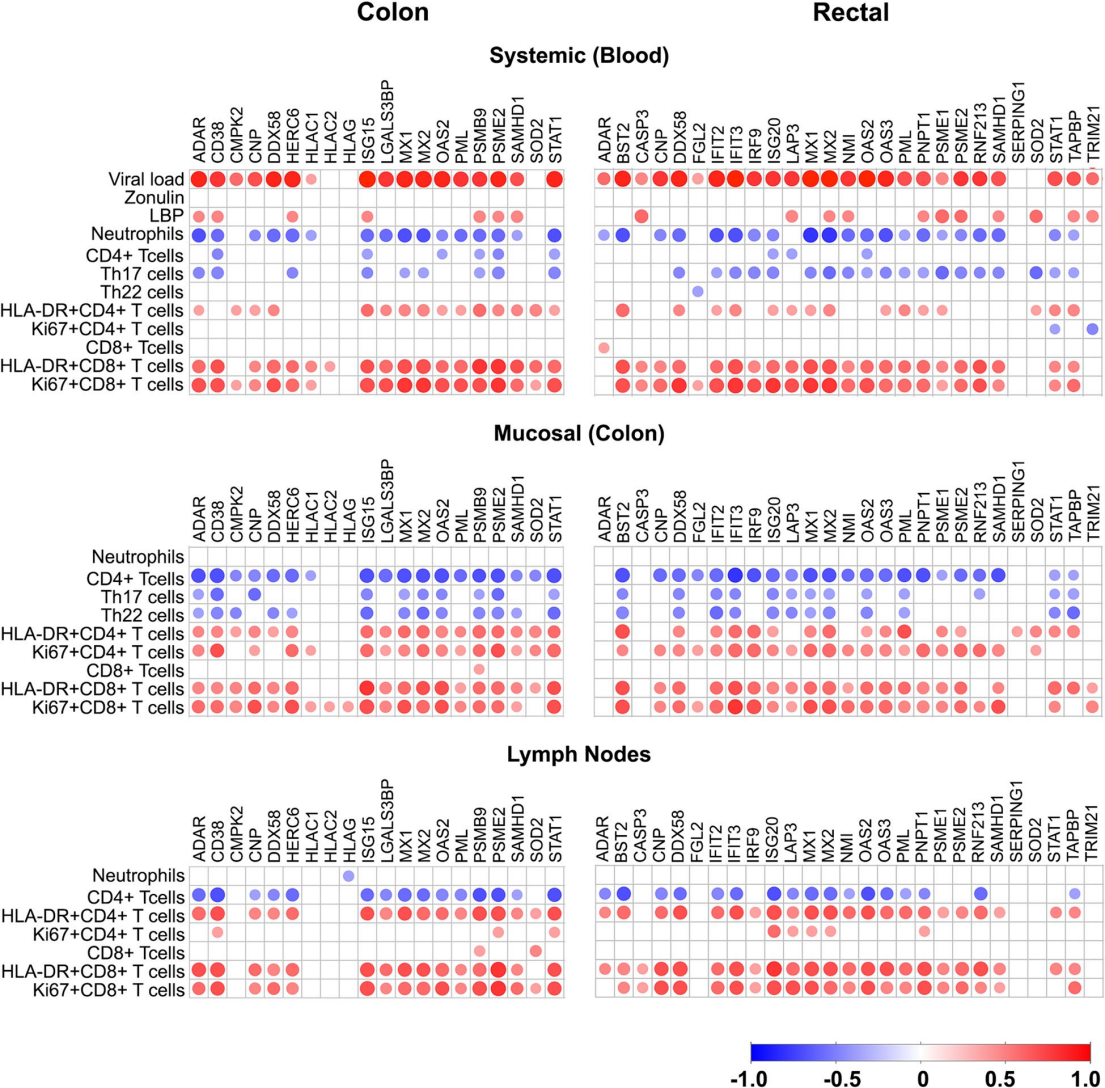
Relationship between IFN pathway proteins in colon and rectal tissues with inflammation and immune activation during acute SIV infection in rhesus macaques. Data shown are Spearman correlation values of normalized protein abundances (log_2_ transformed) to percentages of immune cell counts of total cells. Activation (HLADR) and proliferation (Ki67) markers were also measured in CD4+ and CD8+ cells as a percentage of total CD4+ and CD8+ cells, respectively. Blue denotes inverse correlations, red indicate positive correlations, and white are those that did not reach statistical significance (*p* < 0.05). Proteins encoded by IFN-stimulated genes, such as STAT1, ISG15, MX1, MX2 and OAS2 negatively correlated with the abundance of immune cells but positively with T-cell activation and proliferation markers.

The interferon response has been widely studied in the context of both SIV^10^ and HIV infection^11^. HIV is known to trigger IFN-I by innate immune sensors, eliciting broad anti-viral effects through interferon-simulated genes^12^. However, a complete proteome characterization of the IFN response to SIV/HIV infection has not been previously described. Using GSEA analysis, we identified 17 (colon) and 11 (rectal) proteins in the IFN pathway that significantly increased in response to infection, peaking at 14 dpi with viral load (Figs. 2 and 4). Most of these proteins have previously identified roles in HIV/SIV infection (Adenosine Deaminase RNA Specific, ADAR; Bone Marrow Stromal Cell Antigen 2, BST2; CD38; Cytidine/Uridine Monophosphate Kinase 2, CMPK2; 2′,3′-Cyclic Nucleotide 3′ Phosphodiesterase, CNP; RNA Sensor RIG-I, DDX58; HECT And RLD Domain Containing E3 Ubiquitin Protein Ligase Family Member 6, HERC6; ISG15 Ubiquitin Like Modifier, ISG15; Galectin 3 Binding Protein, LGALS3BP; MX Dynamin Like GTPase 1, MX1; MX Dynamin Like GTPase 2, MX2; 2′-5′-Oligoadenylate Synthetase 2, OAS2; PML Nuclear Body Scaffold, PML/TRIM19; SAM And HD Domain Containing Deoxynucleoside Triphosphate Triphosphohydrolase 1, SAMHD1; Superoxide Dismutase 2, SOD2; Signal Transducer And Activator Of Transcription 1, STAT1; TAP Binding Protein, TAPBP; Tripartite Motif Containing 21, TRIM21). Proteasome Activator Subunit 2 (PSME2) has been identified in a gene expression analysis in HIV-associated neurocognitive disorder, but has no known role in HIV pathogenesis^13^. Both Proteasome 20S Subunit Beta 9 (PSMB9, immunoproteasome) and Fibrinogen Like 2 (FGL2) are novel proteins identified in the context of HIV infection. Over expression of FGL2 has prothrombinase activity and is known to induce EMT^14^.

We next compared the relationship between IFN-proteome changes to the cellular immune response. This included a correlation analysis between protein abundance and the percentage of immune cell populations (Fig. 4). Activated CD4+ and CD8+ T cells, as a percentage of total CD4+ and CD8+ T cells, were positively associated with the IFN-proteome signature in all compartments including the blood, colon, and lymph node. Total CD4+ T-cells, Th17 cells, and Th22 cells negatively associated with IFN-proteome signature, more pronounced in the colon and rectum, consistent with the depletion kinetics of CD4+ T cells during SIV infection^15^. Neutrophil infiltration into infected mucosal tissues is a hallmark of the innate immune response^16^, and while blood neutrophils (measured as a percentage of total CD45+ cells) negatively associated with the IFN-proteome signature there were no associations to mucosal neutrophils. However, as we did not measure other neutrophil markers we could not make further comparisons to neutrophil phenotype or activation status.

Epithelial to mesenchymal transition (EMT) is a transient process where epithelial cells transition to mesenchymal cells either partially or fully, and is characterized mostly by a structural/phenotypical change of the cell^17^. A metastable cell having both epithelial and mesenchymal traits has been observed in some studies, and highlights the plasticity of the process^18^. EMT is observed during wound healing, when morphological changes to the epithelial cells is needed to allow migration of cells across the wound for closure. However, unchecked EMT processes can lead to fibrotic diseases, inflammatory diseases, and cancer^18^. During EMT there is a loss in epithelial markers including cell–cell junctions and a reorganization of cellular cytoskeleton structure. The amount of reorganization differs depending on the extent of change and tissue cell type. Certain protein expression patterns, such as the decrease in classical epithelial markers E-cadherin, claudins, occludin, desmoplakin and keratins as well as an increase in mesenchymal markers collagens, N-cadherin and fibronectin, are classical phenotypical changes during this process^18^.

**Figure 5:**
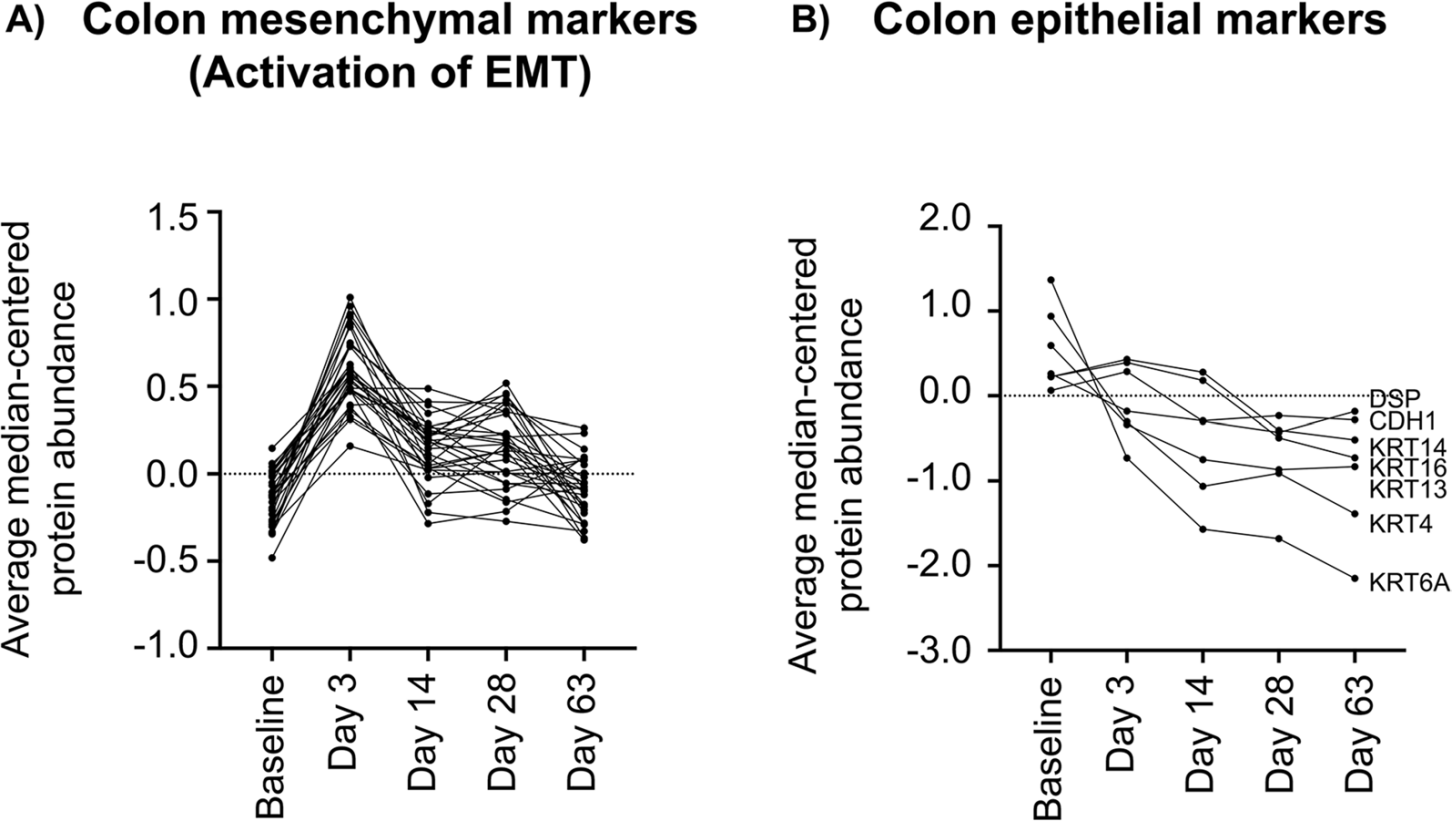
Proteins involved in epithelial to mesenchymal transition (EMT) pathway are altered in the colon in early SIV infection. Average abundance of epithelial and mesenchymal protein biomarkers are plotted over the course of SIV infection. (**A**) Proteomic expression of colon tissues pre- and post-SIV infection showed an increase in mesenchymal markers (collagens) as well as signaling markers that are known to induce EMT starting at Day 3 post-infection. Graphs of each protein, for each animal, are shown in Supplementary Fig. 2. (**B**) A decrease in epithelial markers (E-cadherin, keratins, desmoplankin) was also observed after infection by Day 14.

In this study, we found both mesenchymal and epithelial markers, as well as signaling proteins that induce EMT, significantly altered in colon, but not rectal, tissues at 3 dpi (Fig. 5). Up-regulation (Fig. 5a, Supplementary Fig. 2) of mesenchymal structural markers was observed at 3 dpi including collagens, vimentin (VIM), Fibrillins (FBN1, 2), ABI Family Member 3 Binding Protein (ABI3BP), Laminin (LAMC1) and nidogen (NID2). Signaling proteins that activate or regulate EMT, including Biglycan (BGN), Caldesmon 1 (CALD1), Decorin (DCN), LDL Receptor Related Protein 1 (LRP1), Lumican (LUM), Procollagen-Lysine,2-Oxoglutarate 5-Dioxygenase 3 (PLOD3), Secretogranin 2 (SCG2), Transforming Growth Factor Beta 1 (TGFb1), Transglutaminase 2 (TGM2), and Tenascin C (TNC) were also increased by 3 dpi. Following activation of EMT, epithelial structural markers are known to either change location on the cells (losing polarity), or even decrease, as the phenotype of the cells change from an epithelial to a mesenchymal state. At 14 dpi, many epithelial markers were decreased in comparison to baseline (Fig. 5b) including E-cadherin (CDH1), Desmoplakin (DSP), and many keratins. A decrease of E-cadherin is the prototypical signature of EMT^19^. There was no similar EMT signature observed in the rectal tissues.

### Associations of EMT and IFN signatures during acute SIV

Soluble epithelial dysfunction biomarkers were measured in the blood, including zonulin and lipopolysaccharide binding protein (LBP). A breakdown in the intestinal barrier releases zonulin into the bloodstream and is a biomarker of diseases such as diabetes, celiac disease and obesity. Measuring LBP is an indirect method of determining microbial translocation and is used to describe sepsis. Interestingly, we did not observe a correlation with most of the epithelial or mesenchymal proteins and zonulin in either the colon or the rectal tissues (Supplementary Figs. 3, and 4). It may be that early events in the colon are not observable in the systemic compartment. Only a few EMT markers in the colon correlated with LBP, including desmoplakin and Collagen Type XII Alpha 1 Chain (COL12A1, inverse correlation), as well as Keratin 82 (KRT82) and Procollagen-Lysine,2-Oxoglutarate 5-Dioxygenase 3 (PLOD3) (positive correlation).

Neutrophils are thought to infiltrate tissues and cause barrier damage during clearance of infections^20^. In previous studies, neutrophils have been shown to contribute to the activation of EMT and can promote tumor migration through IL17a^21^. Neutrophils in the blood positively correlated with epithelial keratins 13, 3, 4, 5 and 6A (Supplementary Fig. 3a) but had no correlation to EMT markers in the colon tissues.

As T-cells play a large role in HIV infection, levels of CD4+ T-cells (including Th17 and Th22 cells) in the blood, colon and lymph nodes were measured, along with activation (HLADR) and proliferation (Ki67) markers. Th17 cells have well characterized roles in both immune pathology as well as maintaining homeostasis at mucosal barrier sites. Th22 cells also enhance immune responses to pathogen infection while promoting repair of damaged epithelial barriers^22^. In our study, Th17 cells in both the colon and blood, and Th22 cells in the colon, were seen to have a positive correlation with keratin expression in colon tissue. Like neutrophils, Th17s produce IL17 and may induce EMT expression. However, a decrease in Th17 cells after infection and a negative correlation with IL17 and colon markers of EMT (Supplementary Fig. 3c), suggests that Th17 cells do no activate EMT in this context.

CD4+ T-cells are targets for HIV infection and a decrease in total CD4+ T-cell counts is a hallmark of HIV infection, which we observed in our study. Colon tissue epithelial markers were positively correlated with the amount of both systemic and mucosal CD4+ T-cells, with more biomarkers associating with CD4+ T-cells in the lymph node (15 proteins) in comparison to both the colon and blood CD4+ T-cells (12 and 7 proteins, respectively). Conversely, the activation and proliferation of T-cells were negatively correlated with epithelial markers, as CD4+ and CD8+ T-cells were activated and started proliferating after infection. A stronger systemic rather than local association with T-cells and epithelial markers was observed, with more proteins correlating to activation and proliferation of T-cells in the lymph node than in the colon (Supplementary Fig. 3). In contrast to the colon, there were very few significant correlations (Keratin 1, KRT1; Keratin 20, KRT20; Plakophilin 2 PKP2) between EMT proteins in the rectal tissues and immune cell abundance or activation (Supplementary Fig. 4). Few mesenchymal or EMT activation signaling proteins were correlated with immune cells, as EMT initiated earlier (3 dpi) than the immune response (14 dpi).

Peak viral load (PVL) during acute HIV infection is associated with the amount of time it takes to progress to AIDS and is therefore a strong marker of pathogenesis. Colon epithelial proteins were inversely correlated with viral load, decreasing by 14 dpi when peak viremia occurred (Supplementary Fig. 3). However mesenchymal markers which started increasing at 3 dpi did not correlate with viral load, indicating that the onset of EMT, characterized by an increase in mesenchymal markers, occurred prior to viral peak. We further explored the relationship between both EMT and IFN proteome alterations and viral load. We used the LASSO algorithm (Least Absolute Shrinkage and Selection Operator) with supervised cross validation by PLSR (Partial Least-Squares Regression) to evaluate the relationship between either EMT or IFN, and viral load.

**Figure 6:**
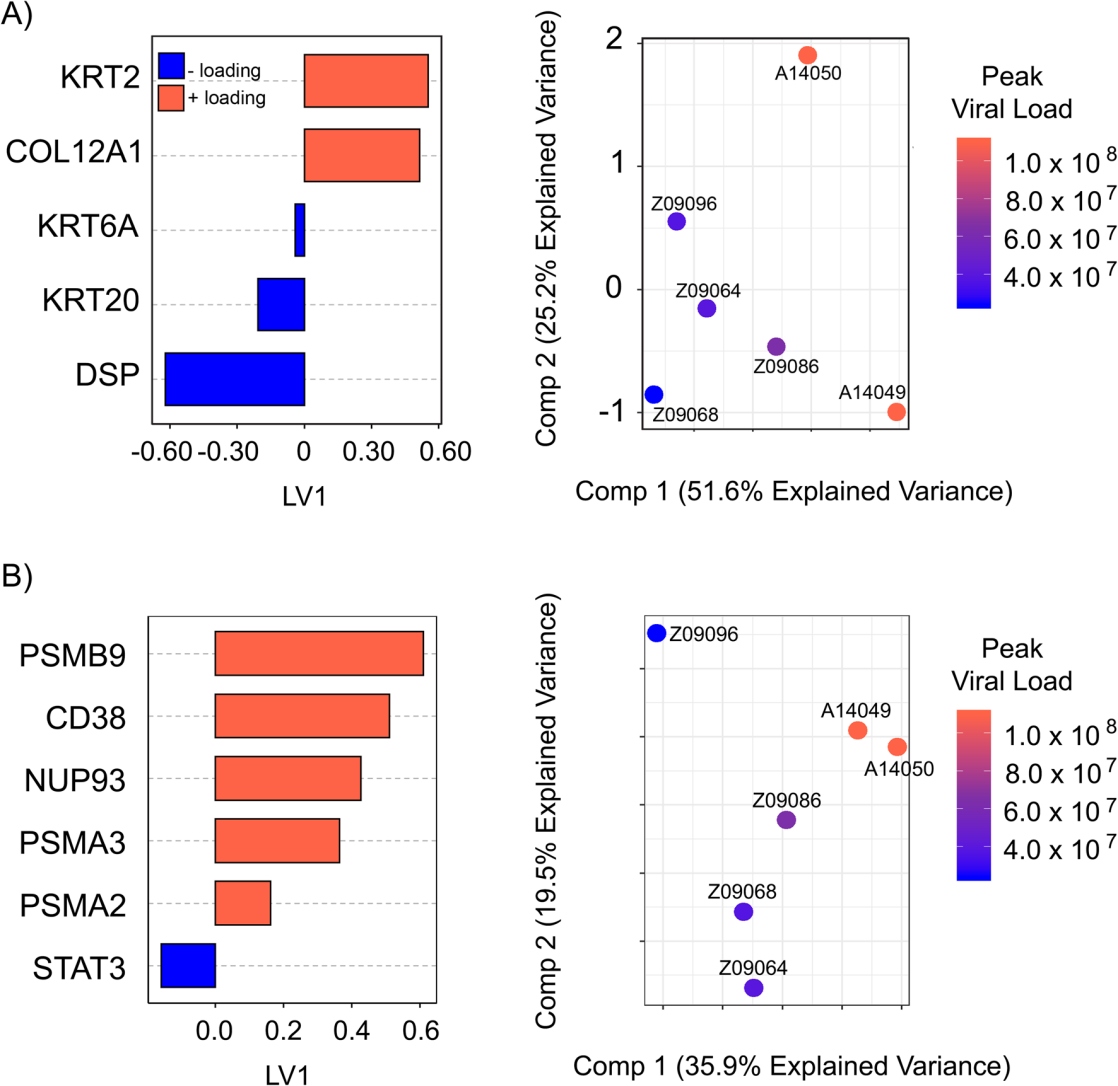
Relationship between peak viral load and EMT or IFN protein biomarkers. Using Least Absolute Shrinkage and Selection Operator (LASSO) method with supervised cross validation by PLSR (Partial Least-Squares Regression) protein signatures for (**A**) EMT or (**B**) IFN were observed to predict peak viral load at Day 14 post-infection. Comparing the changes in EMT proteins from baseline to Day 3, 5 EMT biomarkers were selected with a moderate fit of the data (R2 = 0.754, MSEP = 20.8%, variance explained on PLS component 1 = 51.6%). Comparing the changes in IFN proteins from baseline to Day 3, 6 EMT biomarkers were selected with a good fit of the data (R2 = 0.93, MSEP = 43.9%, variance explained on PLS component 1 = 35.9%).

For the EMT signature, changes in the expression of certain epithelial and mesenchymal proteins after 3 dpi were able to predict PVL with moderate accuracy of 75% (Fig. 6a), (R2 = 0.754, MSEP = 20.8%, variance explained on PLS component 1 = 51.6%). A total of 5 EMT factors were selected in the model, including Keratin 6a (KRT6a) which was highlighted in Fig. 4 to significantly decrease after infection. Other epithelial and mesenchymal markers selected included KRT2, KRT20, COL12A1, and DSP. Together, this indicated a moderate predictive relationship between the degree of changes in EMT proteins early in SIV infection and peak viral load at 14 dpi.

For the IFN signature, changes in the expression of certain interferon proteins after 3 dpi were able to predict PVL with an accuracy of 93% (Fig. 6b), (R2 = 0.93, MSEP = 43.9%, variance explained on PLS component 1 = 35.9%). A total of 6 IFN factors were selected in the model, including GSEA identified IFN proteins PSMB9 and CD38, as well as NUP93, PSMA2, PSMA3 and STAT3. These proteins indicated a good predictive relationship between the initial IFN proteome response and peak viral load.

## Discussion

During acute HIV infection, the gastrointestinal tract is a major site of HIV replication leading to substantial depletion of lamina propria CD4+ T cells^23^. With treatment, there is a suppression of viral replication and a partial restoration of CD4+ T cells, but epithelial barrier damage that occurs due to HIV infection persists^24^. In this study, to the best of our knowledge, we identified new biomarkers of early SIV infection which related to EMT activation signaling and mesenchymal biomarkers at 3 dpi (BGN, CALD1, DCN, LRP1, LUM, PLOD3, SCG2, TGFb1, TGM2 and TNC). This was followed by a decrease in epithelial biomarkers at 14 dpi which was associated with the depletion of CD4+ T cells as well as immune cell activation and viral load. In addition, this analysis identified new proteins involved in the antiviral immune response (PSMB9 and FGL2) during peak viral load. Collectively this information adds to our understanding of HIV infection by identifying new proteome biomarker changes in early infection related to structural epithelial barrier integrity, which may be important for severity of disease progression and immune activation.

Neutrophils, the first responders during infection, help control pathogenic infections by triggering an inflammatory response, but also damage tissue due to the release of reactive oxygen species (ROS), proteases or other harmful molecules^25^. In our study, peripheral neutrophils negatively associated with protein markers of EMT and IFN response in the colon tissue. However, mucosal neutrophils were not associated with these signatures. Our previous analysis showed that neutrophils did not infiltrate the tissues after infection^6^, therefore an association was not expected. However, neither neutrophil function (such as releasing ROS) nor phenotype was captured in our assays and it is possible these relationships may be important and a future area of investigation.

The EMT process is characterized by the disassembly of epithelial cell–cell contacts and a loss of polarity^17^ to provide epithelial cells plasticity and a motile mesenchymal phenotype^26^. The repression of these epithelial genes and restructuring of the cells are simultaneously coupled to activation of mesenchymal genes, such as vimentin, N-cadherin and collagens^17^ to enable this process. EMT is essential for tissue development as well as wound healing, and the reverse process, mesenchymal-to-epithelial transition (MET), is important for final development of cellular differentiation^27^. Though a transient EMT response is essential for the wound healing process allowing for re-epithelialization and extracellular matrix remodeling, unchecked or sustained EMT activation leads to a pathologic wounding response causing tissue fibrosis, inflammation and even cancer metastasis. It has been shown that a continued EMT response occurs through inflammation, specifically IFN^28^, leading to eventual organ destruction. For example, the intestinal fibrosis observed during inflammatory bowel diseases (IBD) is considered the final outcome of the host reaction to persistent inflammation^29^ and occurs through EMT. Our results from this study indicate that follow up studies targeting MET may be warranted as avenues of research to augment treatment regimens such as ART to aid in wound healing from gut disfunction.

It has been previously shown that HIV infection causes EMT in the kidneys of a mouse model, leading to HIV-associated nephropathy and compromised barrier function^30^. As well, a recent paper has shown that HIV-1 proteins gp120 and tat were able to induce EMT in oral and genital mucosal cells in vitro^31^. EMT has been proposed as a mechanism of HIV-induced carcinogenesis in mice^32^, and has been recently proposed to play a role in the sequestration of virions in mucosal epithelial cells^33^. Whether HIV can induce this pathway in intestinal cells by direct viral-host interaction in humans, or if EMT can influence HIV pathogenesis, has yet to be studied. Wound healing ability in African green monkeys has been recently shown to contribute to SIV pathogenesis control^8^, indicating that unchecked or absent wound healing processes may underlie SIV and HIV pathogenesis that is observed in rhesus macaques and humans, respectively.

In the context of acute wound healing and inflammatory injury, EMT is triggered by numerous growth factors, chemokines and MMPs^34^. Interferons, in particular have been linked to EMT activation in the context of cancer ^35^ and both TNFa and IFNg have both shown to induce EMT in vitro^36^. The roles of IFN in HIV infection have been extensively studied, and evidence to support a link between a continued IFN response and HIV pathogenesis has been discovered by investigating mechanisms by which natural monkey hosts do not develop AIDS^37,38^. Our proteome analysis of SIV-infected rhesus macaques showed IFN was activated in both colon and rectal tissues after infection correlating with peak viral load. Many proteins encoded by interferon-stimulated genes that we identified have been previously observed in HIV infection including STAT1, MX1, MX2, OAS2, however, PSMB9 and FGL2 are novel. PSMB9 contributes to autoinflammatory syndromes caused by loss of function mutations and accompanied by a type I interferon signature^39^. FGL2 has been identified as a novel effector molecule of Treg cells playing a critical role in innate and adaptive immunity^40^. The functions of these novel proteins warrant further investigation to a possible role in IFN-related HIV pathogenesis.

The limitation of this study was the use of a small animal group and the inability to collect samples from a control group (one with no SIV infection). Without a mock challenge we are limited in determining how repeated sedation and biopsies may have impacted the data. However, we have tried to improve on this limitation by collecting multiple baseline timepoints and averaging the results for our analysis. Furthermore, proteomic analysis was restricted to whole tissue and thus contributions of these changes to different cell populations was restricted to correlative analyses.

Our results indicate that a more activated CD4+ and CD8+ T cell environment, in addition to a loss in barrier integrity through EMT activation, could lead to an increase in viral load since more viral particles may be able to penetrate the ‘leaky’ tissues and reach activated target T-cells. As well, immune cells and cells expressing a more mesenchymal phenotype have been shown to reciprocally activate each other in tumor cells^41^. These events could exacerbate the known vicious cycle of HIV pathogenic events in the gut tissue, where barrier damage leads to systemic exposure to gut microbial products and increasing T-cell activation.

In conclusion, this analysis has identified new biomarkers of early SIV infection in the gut tissue proteome related to EMT activation, the IFN response, immune activation and inflammation. This provides further data on early protein biomarkers and pathways that may be important for contributing mechanisms for HIV infection and pathogenesis. In the case of HIV infection, follow-up research on the relationship and role of these biomarkers in HIV pathogenesis is warranted to determine if these proteins are contributors to disease pathogenesis in chronic HIV infection.

## Methods

### Study animals

Animals were housed and cared for in Association for the Assessment and Accreditation of Laboratory Animal Care international (AAALACi) accredited facilities, and all animal procedures, methods, and experimental protocols were performed according to protocols approved by the Institutional Animal Care and Use Committee (IACUC) of University of Washington. All methods were carried out in accordance with relevant guidelines and regulations. All methods are reported in accordance with ARRIVE guidelines for the reporting of animal experiments. Six adult male rhesus macaques (*Macaca mulatta*) were infected intrarectally with 100,000 TCID50 SIV_MAC239x_. Baseline samples for blood, lymph node, rectal and colon biopsies were taken 56- and 21-days pre-infection (for control samples), and 3, 14, 28 and 63 days post-infection as previously described^42^ (Fig. 1). Viral loads were determined by real-time reverse transcription (RT)-PCR using primers specific for SIV*gag* as previously described^43^. ELISAs were read using an iMark Microplate Reader (Biorad, Hercules, CA). Animals were euthanized as per the protocol-specified experimental endpoint between 101 and 141 days post-infection.

### Sample preparation for mass spectrometry

Proteins were extracted from frozen colon biopsies as previously described^44^. Frozen tissue samples were homogenized, centrifuged, and digested for mass spectrometry as previously described^44^. Briefly 700 µl of extracted protein was denatured with urea exchange buffer (8 M prepared in 0.05 M HEPES buffer) for 10 min, washed with urea buffer, treated with 100 µl of iodoacetamide, and incubated. After centrifugation, samples are washed with urea buffer, then HEPES buffer twice. Benzonase solution (in HEPES with MgCl_2_) was added and incubated for 30 min then washed. Proteins were trypsin digested and stored at − 80 °C until mass spectrometry preparation.

### Reversed-phase liquid chromatography

Samples were cleaned using reversed-phase liquid chromatography as previously described^44^. The elution gradient was from 97% buffer A (20 mM ammonium formate) to 70% buffer B (90% acetonitrile, 20 mM ammonium formate) over 35 min at a constant flow rate of 150 µl/min. Cleaned peptides were quantified using LavaPep’s Fluorescent Peptide and Protein Quantification Kit (Gel Company) according to manufacturer’s protocol.

### Mass spectrometry

Equal amounts of peptides for each sample were injected into a nano-flow liquid chromatography system (Easy nLC, Thermo Fisher) connected inline to a Q Exactive Plus Quadrupole Orbitrap mass spectrometer. The elution gradient was from 2% buffer A to 30% buffer B in 120 min at a constant flow rate of 200 nl/min. MS spectra were acquired on the Orbitrap analyzer at 70,000 resolution at 200 m/z. Raw MS spectra were processed by Progenesis QI software (v2.0, Nonlinear Dynamics) and Mascot (Matrix Science) as previously described^44^. Technical variability was determined by the addition of a protein mix of all the samples. Search results were entered into Scaffold (v4.4.1.1; Proteome Software, Portland, OR) to determine protein identifications (80% peptide confidence; 95% protein confidence, with minimum of 2 unique peptides per protein).

### Statistical analysis

Normalized protein abundance values were generated with Progenesis, outliers with a median normalized abundance greater than one standard deviation removed. Proteins with high technical variance among standards (Coefficient of variance > 25%) were removed from downstream analysis. Protein differences were determined using paired, non-parametric Mann–Whitney tests, comparing average baseline values to each time point post-infection (3, 14, 28, 63 dpi). Multiple hypothesis testing correction was performed using the Benjimani–Hochberg method (false discovery rate, FDR = 5%). Hierarchical cluster analysis (NMF package in R v3.6.1) was performed on proteins differentially abundant at any time point, using Spearman rank correlation as the distance metric. Differentially regulated proteins were used to characterize top biological pathways and functions altered during acute infection, using both DAVID (Database for Annotation, Visualization, and Integrated Discovery, v6.8) and IPA (Ingenuity® Pathway Analysis) software. Multivariate models were performed to determine a minimum EMT/IFN protein signature needed to distinguish indicating variables of SIV pathogenesis: peak viral load (14 dpi). Models were constructed using the LASSO method for regression shrinkage and selection using glmnet in R (K-fold cross validation). PLSR assessed the ability of LASSO features describe variance in either peak viral load using fivefold cross-validation repeated 50 times with 2 components selected after tuning, according to the MixOmics package in R (mixOmics v6.6.2). Model fit (R2), mean squared error of prediction (MSEP) and variance explained along PLS components were used to assess predictive ability of the models. Principal component (PC) analysis of normalized LASSO markers was performed using the base package in R.

### Flow cytometry analysis

Flow cytometry staining and analysis was performed as previously described, including the full gating strategy used for this analysis^6^. Briefly, samples were stained with LIVE/DEAD Fixable Aqua Dead Cell Stain (ThermoFisher) and stained with surface antigen markers, or intracellular cytokine staining was performed with permeabilization using Cytofix/Cytoperm (BD Biosciences). Stained samples were fixed in 1% paraformaldehyde and collected on a LSR II (BD Biosciences, La Jolla, CA). Analysis was performed in FlowJo (version 9.7.6, Treestar Inc., Ashland, OR). Neutrophils and CD4+ T cells were measured as a percentage of total CD45 cells. Th17 and Th22 cells were measured as a percentage of total CD4+ T cells. HLA-DR+ and Ki67+ cells were measured as a percentage of total CD4+ or CD8+ cells.

## Supplementary Information

Supplementary Information.

Supplementary Figures.

Supplementary Table 1.

Supplementary Table 2.

## Data Availability

All data generated and/or analyzed during the current study are provided as supplementary information files.
