# Supplementary material for: Mass spectrometry analysis of gut tissue in acute SIV-infection in rhesus macaques identifies early proteome alterations preceding the interferon inflammatory response
Source: Sci Rep. 2023 Jan 13;13:690. doi: 10.1038/s41598-022-27112-y (PMC9839751; doi:10.1038/s41598-022-27112-y)
Supplement: Supplementary file 1 — Supplementary Information. [file 41598_2022_27112_MOESM1_ESM.docx]

**Methods**

*Study Animals*

Animals were housed and cared for in Association for the Assessment and Accreditation of Laboratory Animal Care international (AAALACi) accredited facilities, and all animal procedures were performed according to protocols approved by the Institutional Animal Care and Use Committee (IACUC) of University of Washington. Six male rhesus macaques (*Macaca mulatta*) were infected intrarectally with 100,000 TCID SIV_MAC239x_. Baseline samples for blood, lymph node, rectal and colon biopsies were taken 56 and 21 days pre-infection, and 3, 14, 28 and 63 days post-infection as previously described(*1*). Viral loads were determined by real-time reverse transcription (RT)-PCR using primers specific for SIV*gag*. ELISAs were read using an iMark Microplate Reader (Biorad, Hercules, CA).

*Sample preparation for mass spectrometry*

Proteins were extracted from frozen colon biopsies as previously described(*2*). Frozen tissue samples were homogenized, centrifuged and digested for mass spectrometry as previously described(*2*). Briefly 700µl of extracted protein was denatured with urea exchange buffer (8M prepared in 0.05M HEPES buffer) for 10 minutes, washed with urea buffer, treated with 100µl of iodoacetamide, and incubated. After centrifugation, samples are washed with urea buffer, then HEPES buffer twice. Benzonase solution (in HEPES with MgCl_2_) was added and incubated for 30 minutes then washed. Proteins were trypsin digested and stored at -80^o^C until mass spectrometry preparation.

*Reversed-phase liquid chromatography*

Samples were cleaned using reversed-phase liquid chromatography as previously described(*2*). The elution gradient was from 97% buffer A (20mM ammonium formate) to 70% buffer B (90% acetonitrile, 20mM ammonium formate) over 35 minutes at a constant flow rate of 150µl/min. Cleaned peptides were quantified using LavaPep’s Fluorescent Peptide and Protein Quantification Kit (Gel Company) according to manufacturer’s protocol.

*Mass spectrometry*

Equal amounts of peptides for each sample were injected into a nano-flow liquid chromatography system (Easy nLC, Thermo Fisher) connected inline to a Q Exactive Plus Quadrupole Orbitrap mass spectrometer. The elution gradient was from 2% buffer A to 30% buffer B in 120 minutes at a constant flow rate of 200 nl/min. MS spectra were acquired on the Orbitrap analyzer at 70,000 resolution at 200m/z. Raw MS spectra were processed by Progenesis (Nonlinear Dynamics) and Mascot (Matrix Science) as previously described(*2*). Technical variability was determined by the addition of a protein mix of all the samples. Search results were entered into Scaffold (v4.4.1.1; Proteome Software, Portland, OR) to determine protein identifications (80% peptide confidence; 95% protein confidence, with minimum of 2 unique peptides per protein).

*Statistical analysis*

Normalized protein abundance values were generated with Progenesis, outliers with a median normalized abundance greater than one standard deviation removed. Proteins with high technical variance among standards (Coefficient of variance >25%) were removed from downstream analysis. Protein differences were determined using paired, non-parametric Mann-Whitney tests, comparing average baseline values to each time point post-infection (3, 14, 28, 63dpi). Multiple hypothesis testing correction was performed using the Benjimani-Hochberg method (false discovery rate, FDR=5%). Hierarchical cluster analysis (NMF package in R v3.6.1) was performed on proteins differentially abundant at any time point, using Spearman rank correlation as the distance metric. Differentially regulated proteins were used to characterize top biological pathways and functions altered during acute infection, using both DAVID (Database for Annotation, Visualization, and Integrated Discovery, v6.8) and IPA (Ingenuity^®^ Pathway Analysis) software. Multivariate models were performed to determine a minimum EMT/IFN protein signature needed to distinguish indicating variables of SIV pathogenesis: viral load set point (14dpi). Models were constructed using the LASSO method for regression shrinkage and selection using glmnet in R (K-fold cross validation). PLSR assessed the ability of LASSO features describe variance in either viral load set point using 5-fold cross-validation repeated 50 times with 2 components selected after tuning, according to the MixOmics package in R (mixOmics v6.6.2). Model fit (R2), mean squared error of prediction (MSEP) and variance explained along PLS components were used to assess predictive ability of the models. Principal component (PC) analysis of normalized LASSO markers was performed using the base package in R.

1. C. Moats *et al.*, Antimicrobial prophylaxis does not improve post-surgical outcomes in SIV/SHIV-uninfected or SIV/SHIV-infected macaques (Macaca mulatta and Macaca fascicularis) based on a retrospective analysis. *PLoS One* **17**, e0266616 (2022).

2. K. Birse *et al.*, Molecular Signatures of Immune Activation and Epithelial Barrier Remodeling Are Enhanced during the Luteal Phase of the Menstrual Cycle: Implications for HIV Susceptibility. *J Virol* **89**, 8793-8805 (2015).
