# Supplementary material for: Mass spectrometry analysis of gut tissue in acute SIV-infection in rhesus macaques identifies early proteome alterations preceding the interferon inflammatory response
Source: Sci Rep. 2023 Jan 13;13:690. doi: 10.1038/s41598-022-27112-y (PMC9839751; doi:10.1038/s41598-022-27112-y)
Supplement: Supplementary file 2 — Supplementary Figures. [file 41598_2022_27112_MOESM2_ESM.pdf]

Supplementary Figures:

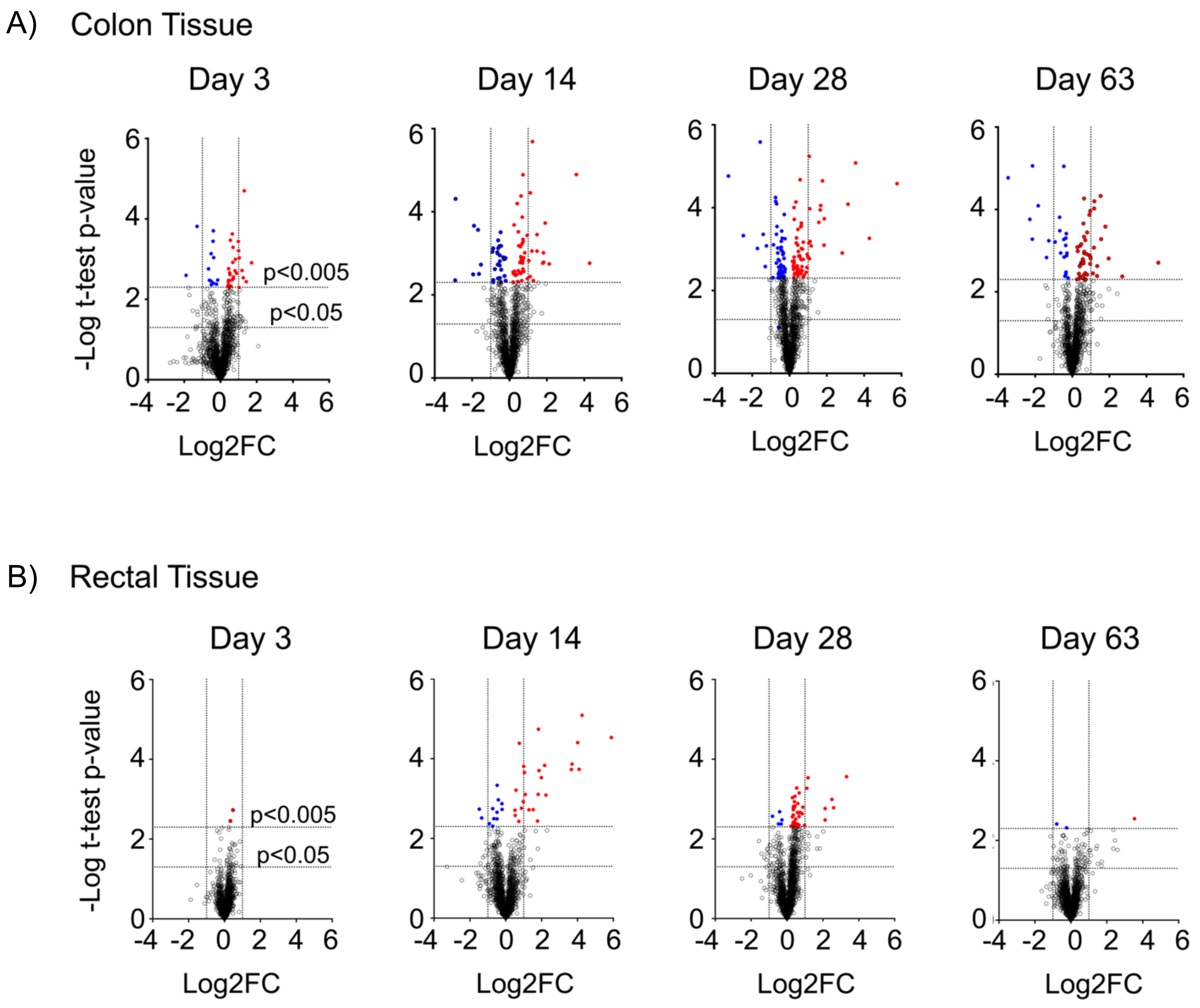

Supplementary Figure 1. Colon and rectal tissue proteome changes post-SIV infection in rhesus macaques. Volcano plots show the protein differences compared to baseline at each time point (day 3, 14, 28, 63) post-SIV infection. More pronounced proteome changes (as shown on y-axis) were observed post-infection in colon tissues compared to rectal tissues, denoted by the number of proteins significantly different after infection. In both compartments, at peak viral load (day 14) there is a similar number of up and down regulated proteins. However, at the other time points evaluated, there is up to a 4-fold greater amount of up regulated proteins in comparison to down regulated.

GSEA EMT protein changes from baseline to day 3

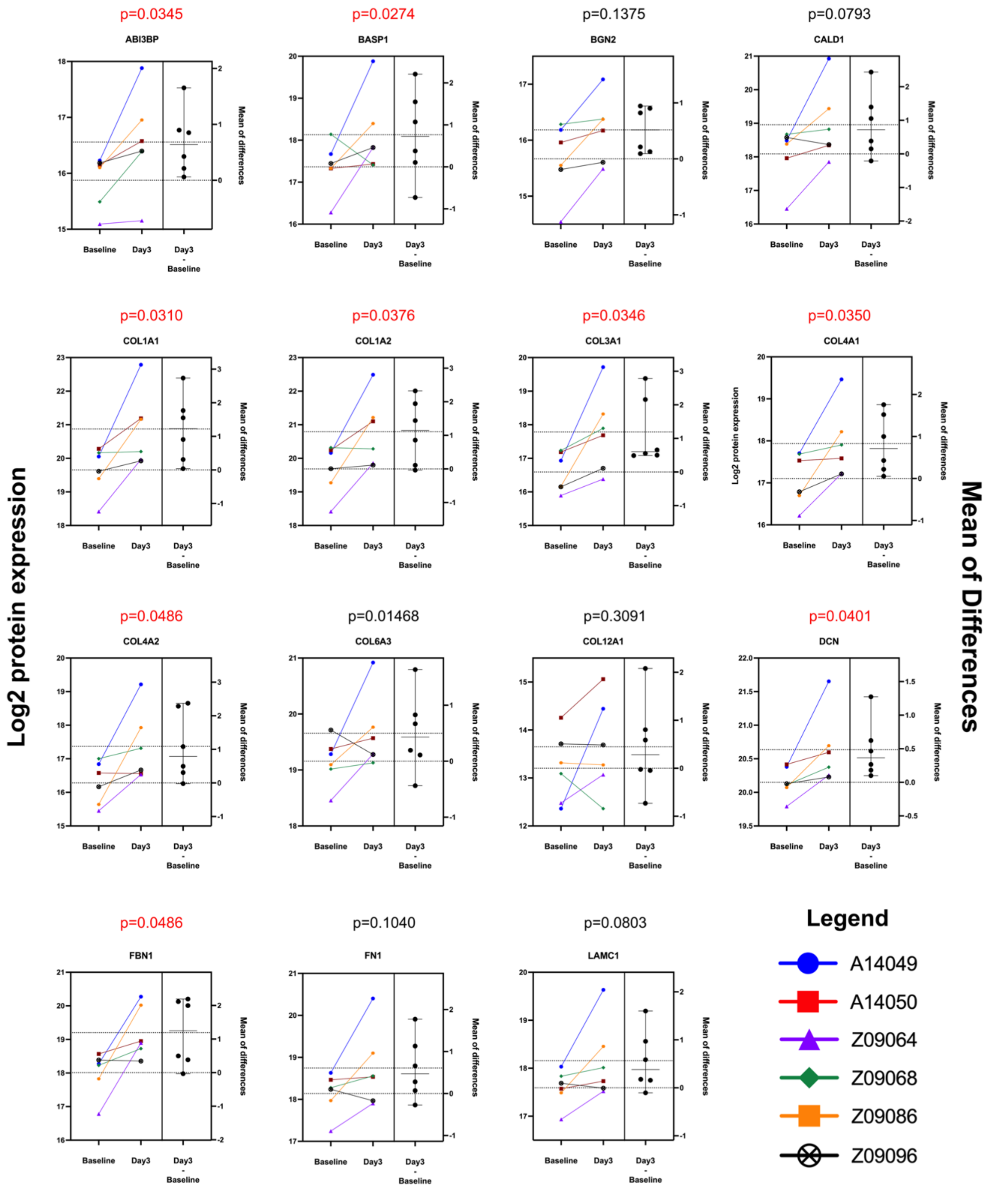

Supplementary Figure 2. Protein expressions at baseline and Day 3 post-infection of signaling proteins that promote activation of EMT (identified using Gene Set Enrichment Analysis (GSEA)), and mesenchymal structural proteins that increase after EMT activation.

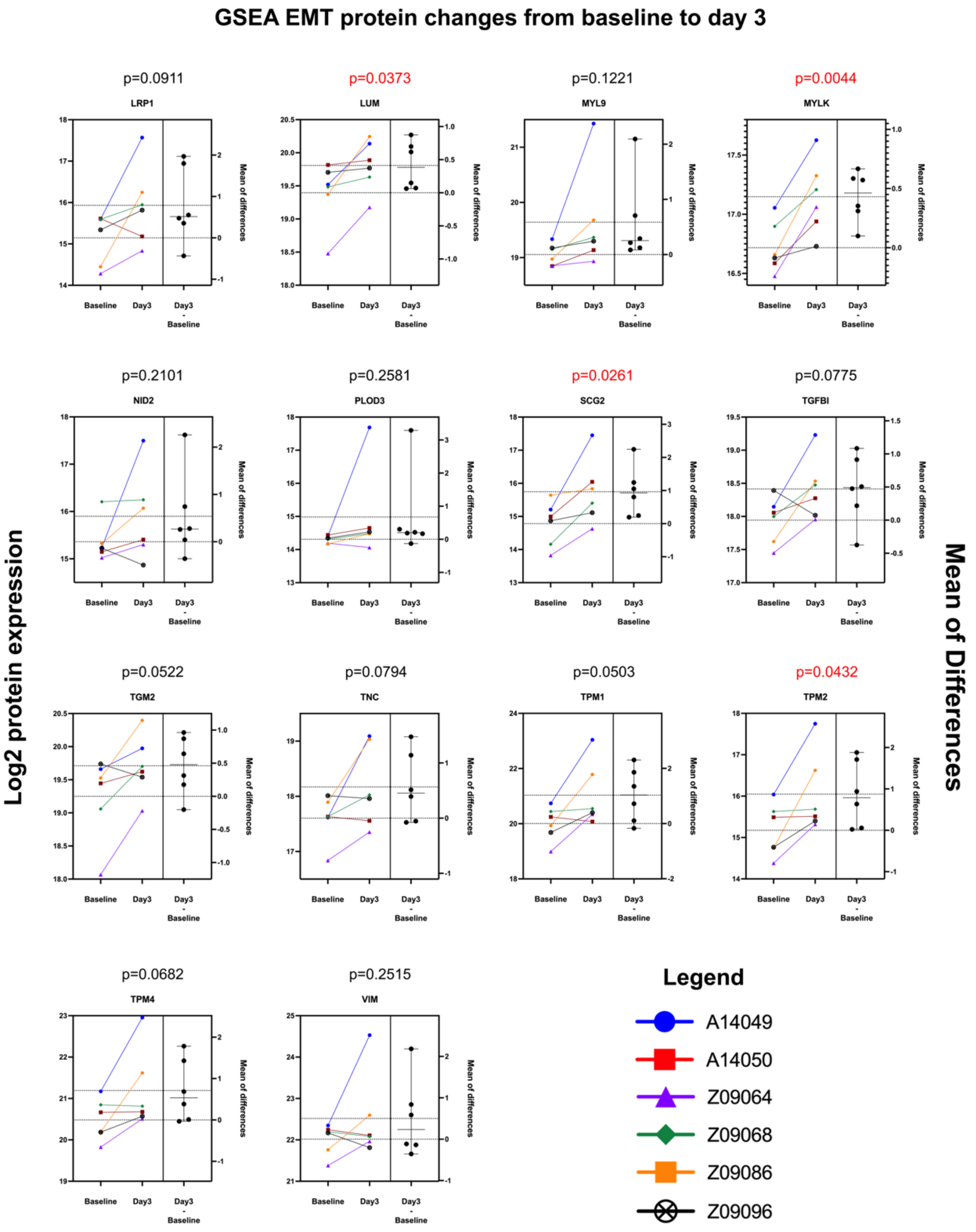

Supplementary Figure 2, cont'd. Protein expressions at baseline and Day 3 post-infection of signaling proteins that promote activation of EMT, and mesenchymal structural proteins that increase after EMT activation. Signaling proteins that activate or regulate EMT, including BGN, CALD1, DCN, LRP1, LUM, PLOD3, SCG2, TGFb1, TGM2 and TNC are increased after infection. Structural mesenchymal proteins including collagens (COL's), vimentin (VIM), Fibrillins (FBN1, 2), ABI3BP, Laminin (LAMC1) and nidogen (NID2) are also increased after infection, indicating the epithelial cells are changing into a mesenchymal phenotype.

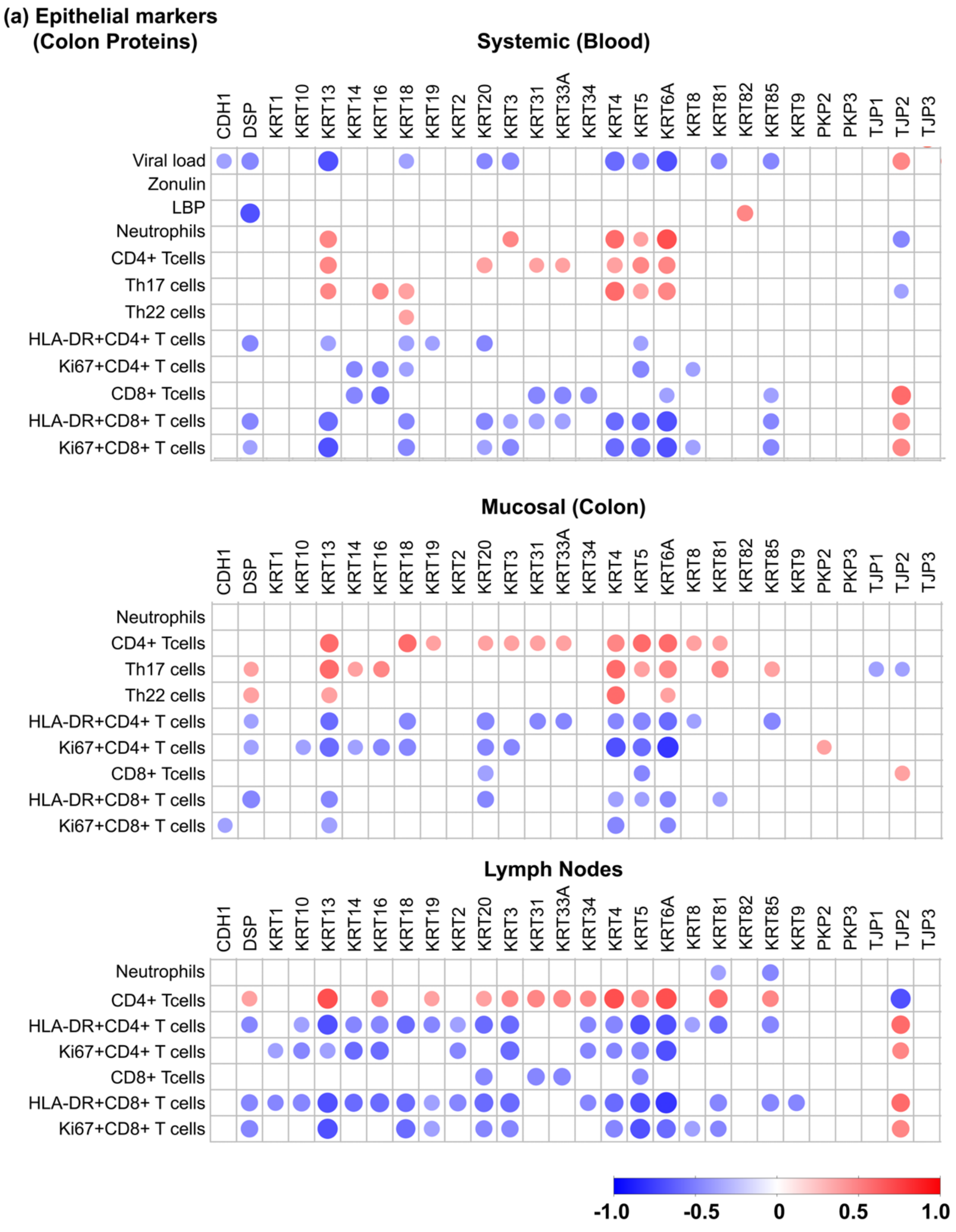

Supplementary Figure 3. Relationship between (a) epithelial protein biomarkers with immune cells during acute SIV infection in colon tissue. A variety of cytokeratins show a positive correlation with the number of CD4+ T cells in the blood, lymph node and colon, but a negative correction with activation and proliferation of CD4+ and CD8+ T cells. The strongest associations included those between Keratins 4, 5 and 6a with viral load, neutrophils in the blood, number of CD4+ T cells, Th17 cells, as well as activation and proliferation of CD4+ and CD8+ T cells.

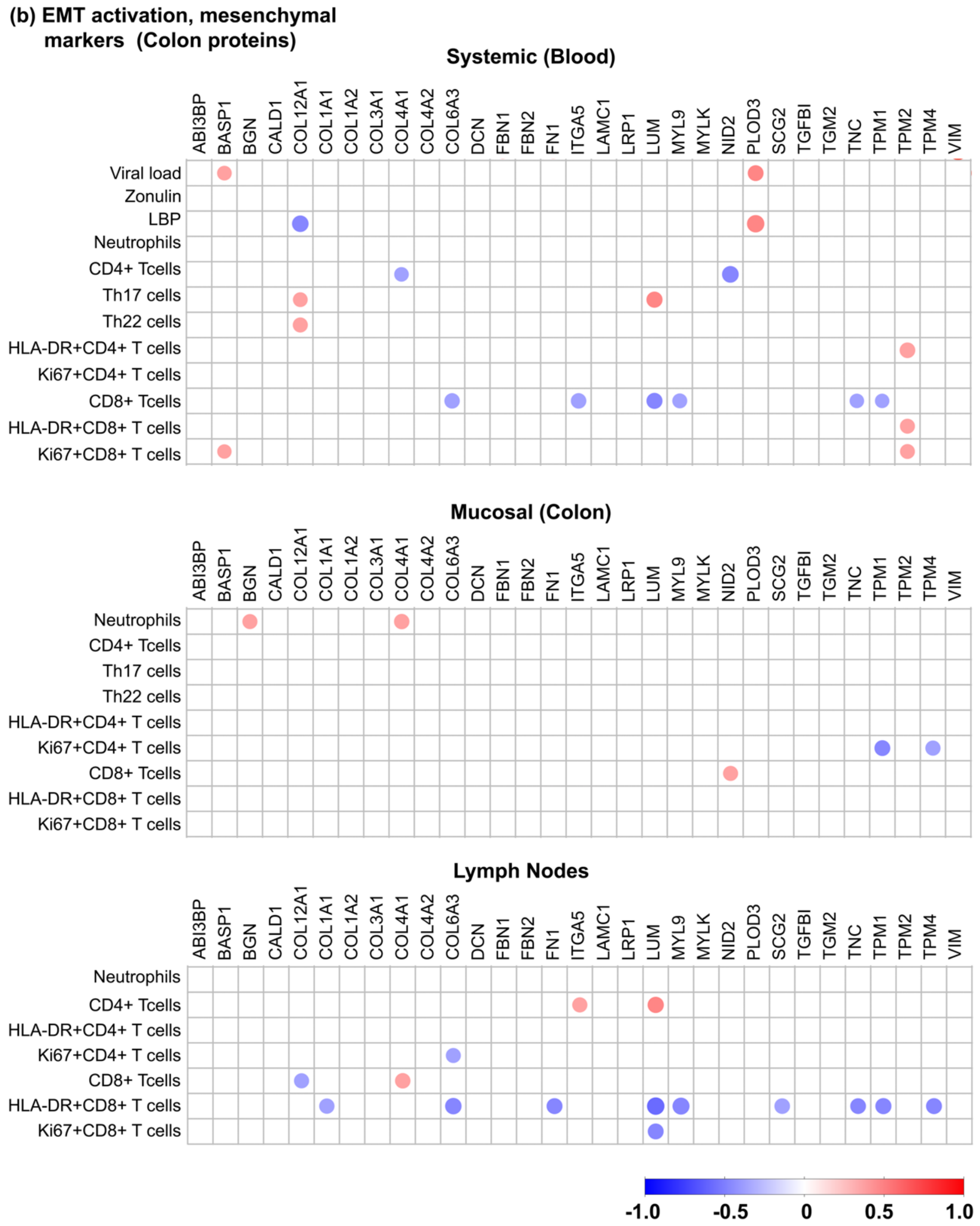

Supplementary Figure 3 cont'd. Relationship between (b) EMT activation signaling proteins and mesenchymal protein biomarkers with immune cells during acute SIV infection in colon tissue. Mesenchymal protein abundance changes occurred early on after infection (Day 3) and therefore did not correlate with the later immune response (day 14).

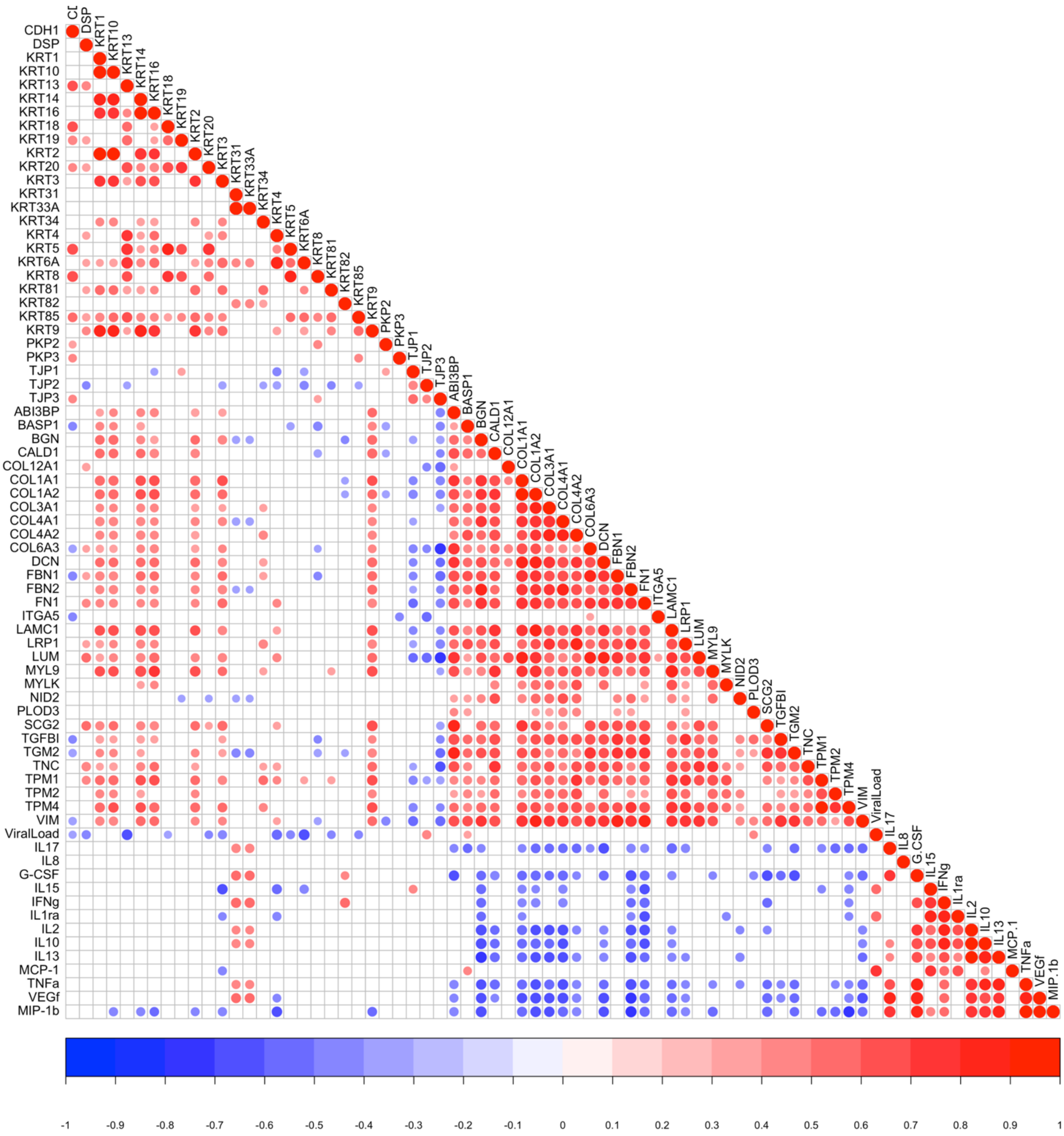

Supplementary Figure 3c. Relationship between colon epithelial and mesenchymal structural proteins as well as EMT activation signaling proteins with cytokines expressed in the blood during acute SIV infection. The expression of mesenchymal phenotype proteins, which increase at day 3, are negatively correlated with many cytokines.

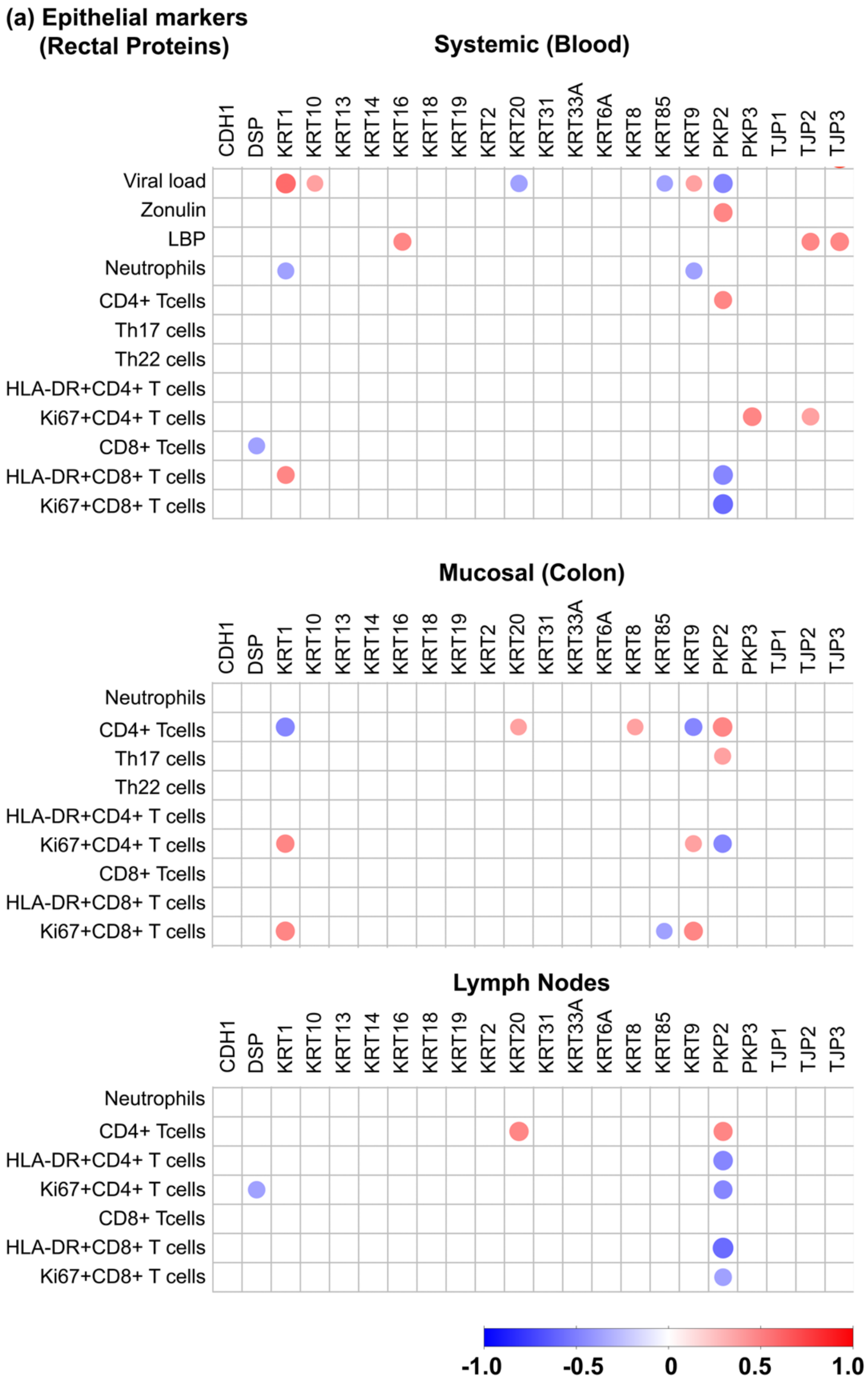

Supplementary Figure 4. Relationship between (a) epithelial and (b) EMT activation signaling proteins and mesenchymal protein biomarkers with immune cells during acute SIV infection in rectal tissue. Blue denotes inverse correlations while red indicate positive correlations. There were only a few correlations between immune marker subtypes and epithelial biomarkers in the rectal tissues.

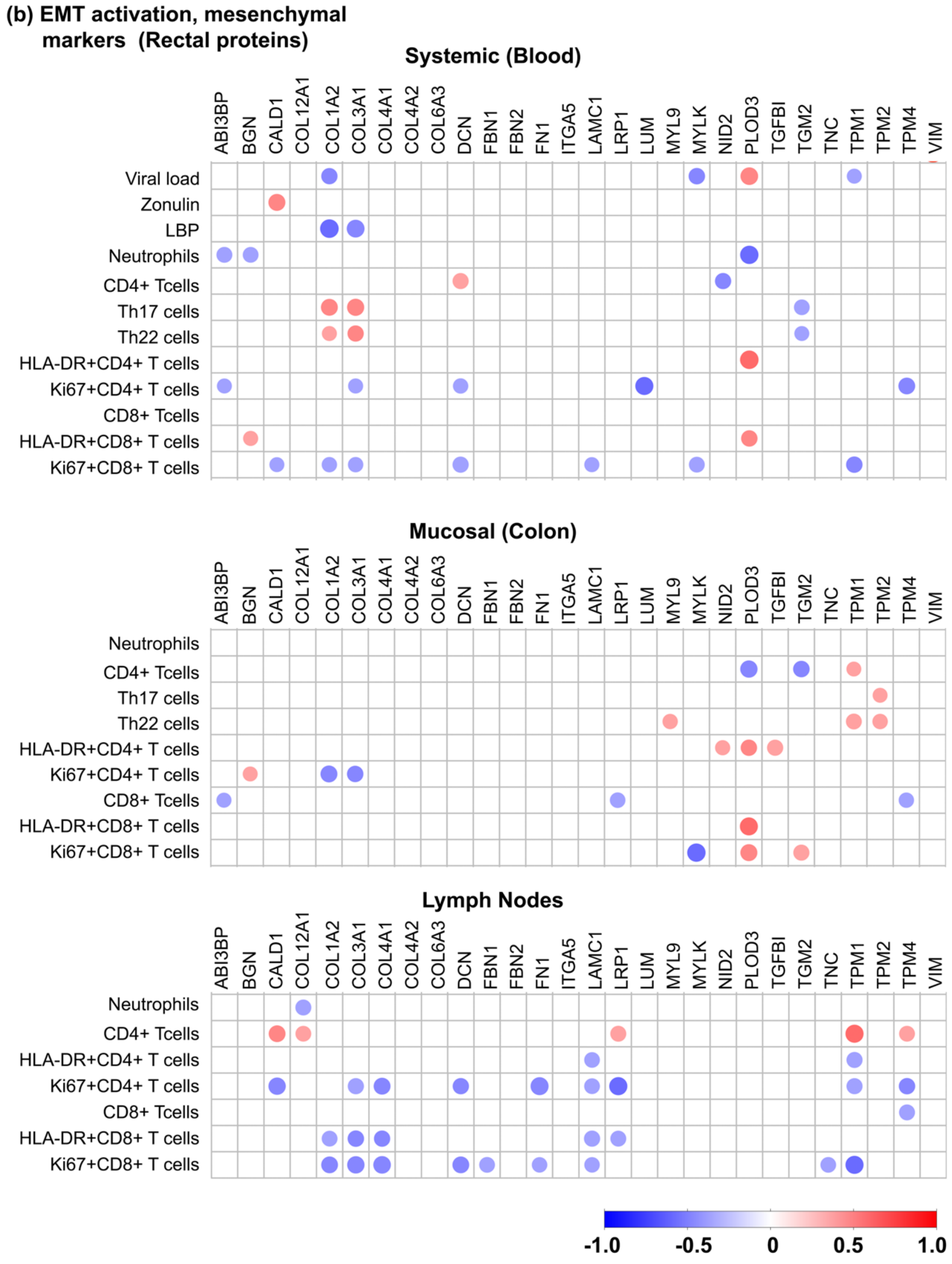

Supplementary Figure 4 cont'd. Relationship between (a) epithelial and (b) EMT activation signaling proteins and mesenchymal protein biomarkers with immune cells during acute SIV infection in rectal tissue. Blue denotes inverse correlations while red indicate positive correlations. There were only a few correlations between immune marker subtypes and EMT biomarkers in the rectal tissues.
